# Epidemiological Impact of a Genital Herpes Type 2 Vaccine for Young Females

**DOI:** 10.1371/journal.pone.0046027

**Published:** 2012-10-11

**Authors:** Yijun Lou, Redouane Qesmi, Qian Wang, Marc Steben, Jianhong Wu, Jane M. Heffernan

**Affiliations:** 1 Mathematics and Statistics, York University, Toronto, Ontario, Canada; 2 Applied Mathematics, The Hong Kong Polytechnic University, Hung Hom, Kowloon, Hong Kong; 3 Ecole Superieure de Technologie, Université Sidi Mohamed Ben Abdellah, Fès, Morocco; 4 Institut National de Santé Publique du Québec, Montréal, Québec, Canada; 5 Centre for Disease Modelling, York Institute for Health Research, York University, Toronto, Ontario, Canada; University of Nebraska – Lincoln, United States of America

## Abstract

Genital Herpes, which is caused by Herpes Simplex Virus-1 or -2 (HSV-1, -2, predominantly HSV-2) is a sexually transmitted infection (STI) that causes a chronic latent infection with outbreak episodes linked to transmission. Antiviral therapies are effective in reducing viral shedding during these episodes, but are ineffective as a whole since many outbreaks are asymptomatic or have mild symptoms. Thus, the development of a vaccine for genital herpes is needed to control this disease. The question of how to implement such a vaccine program is an important one, and may be similar to the vaccination program for Human Papilloma Virus (HPV) for young females. We have developed a mathematical model to describe the epidemiology of vaccination targeting young females against HSV-2. The model population is delineated with respect to age group, sexual activity and infection status including oral infection of HSV-1, which may affect vaccine efficacy. A threshold parameter 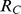, which determines the level of vaccine uptake needed to eradicate HSV-2, is found. Computer simulation shows that an adolescent-only vaccination program may be effective in eliminating HSV-2 disease, however, the success of extinction greatly depends on the level of vaccine uptake, the vaccine efficacy, the age of sexual maturity and safe sex practices. However, the time course of eradication would take many years. We also investigate the prevalence of infection in the total population and in women between 16–30 years of age before and after vaccination has been introduced, and show that the adolescent-only vaccination program can be effective in reducing disease prevalence in these populations depending on the level of vaccine uptake and vaccine efficacy. This will also result in a decrease of maternal-fetal transmission of HSV-2 infection. Another important, if commonsense, conclusion is that vaccination of some females reduces infection in men, which then reduces infection in women.

## Introduction

Genital herpes is one of the most prevalent sexually transmitted infections (STI) in the world. It is estimated that there are 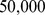 new cases of genital herpes in Canada per year with approximately 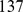 new cases each day [1]. The Centers for Disease Control and Prevention (CDC) estimates that at least 45 million Americans, or one in five adolescents and adults, have genital herpes infection. Genital herpes is caused by either herpes simplex virus type 1 (HSV-1) or type 2 (HSV-2), which are transmissible through skin lesions and mucosa. Once the virus enters a host, it moves from the skin or mucosa of the genitals to the posterior root into the sensory ganglia, where it persists as a latent infection for life. Initial infection results in viral replication in epithelial cells of the genital tract and then spreads into neurons within the dorsal root ganglia in which it remains for life (latency) [2]. Virus is released from the neurons back into the genital tract during reactivations to either a clinically symptomatic or asymptomatic infection and can be further transmitted. HSV-1 is commonly associated with oral infection (herpes labialis or cold sores), but can also cause infection of the genitals through oral-genital sex, even when an oral sore is not apparent. HSV-1 genital herpes is increasing in prevalence [3], however, most genital herpes cases are caused by HSV-2. HSV-2 causes vesicular and ulcerative lesions in adults. Approximately 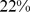 of adults in the USA are HSV-2 positive, approximately 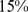 in Europe, as high as 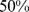 in some developing countries in sub-Saharan Africa, and greater than 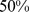 in countries with a large HIV positive population [4]. It is estimated that about 1,640,000 HSV-2 seroconversions occur yearly in the USA [5].

Genital herpes can cause recurrent painful genital sores in many adults, can be severe in people with suppressed immune systems [6], can cause depression [7], can increase the risk of acquisition of HIV [8], [9], and can cause potentially fatal infections or neurological sequelae in newborns from mothers excreting the virus even asymptomatically at the time of delivery [10]. There is therefore an urgent need to consider potential control measures for genital herpes. Antiviral therapies have been shown to be effective in reducing viral shedding, however, since many cases or episodes of genital herpes may be unrecognized (asymptomatic or mild symptoms) antiviral therapies may not be acquired and thus, these drugs can not be effective in controlling HSV transmission in general. Also, if high adherence rates (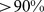) to an antiviral regimen are not maintained, transmission rates can increase [11]. The correct and consistent use of condoms can decrease the risk for HSV-2 acquisition [12]. However, since genital herpes is transmitted by skin-to-skin contact, condoms are not 100 percent protective. Using sexual or other risk factors to screen and identify individuals at higher risks of infection, so as to target interventions, however, may not be feasible or ethically acceptable [13].

Developing an effective HSV-2 vaccine would be a suitable strategy to prevent and control genital herpes infections [4]. Recently, Herpevac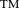 a potential vaccine developed by GSK failed in Phase III clinical trials [14]. Currently, the ImmunoVEX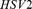 vaccine by Biovex and the ACAM-529 vaccine supported by Sanofi Pasteur are undergoing clinical trials [15], [16]. However, these clinical trials are in very early phases and it is unknown how such vaccines will protect individuals from infection of HSV-2.

Mathematical models of infectious diseases have proven to be a valuable component of public health planning and response. Such models can be employed to evaluate whether a vaccination program will be effective in eliminating a pathogen, or controlling a disease. Such studies can be done prior to the introduction of a vaccine, so as to determine the most effective strategy of vaccination coverage. Mathematical models have been developed to provide insight on the long-term epidemiologic consequences of vaccination against HSV-2 [17]–[21]. However, these models have made some simplifying assumptions which ignore some key characteristics of HSV-2 transmission.

First, they have ignored any differences in HSV-2 transmission or infection by age. The risk of HSV-2 infection is closely related to the sexual behaviour of different age groups. The National Health and Nutrition Examination Survey (NHANES), a series of cross-sectional national surveys from 1976 to 2004 conducted by the National Center for Health Statistics, found that overall HSV-2 seroprevalence rises rapidly in younger age groups and then remains stable among those older than 30 years, in the range of 24 percent to 28 percent prevalence [22], [23]. However, HSV-2 prevalence is negligible among persons who have never been sexually active [24].

Second, except for [19], they have ignored the fact that the risk of acquiring genital herpes also varies by gender. Females are at a greater risk of acquiring genital herpes from male partners than males are from females partners [22], [25]. Seroconversion rates are also much higher for females [26].

Third, some models have focussed on assessing the effectiveness of adult vaccination against HSV-2 [18]–[21]. However, infection with HSV-1 (oral or genital) may prove an HSV-2 vaccine ineffective [19], [27]. This causes a major problem since HSV-1 is highly prevalent as an oral infection in adults [23], [28]. Thus, it may be reasonable to target the vaccination program at children where HSV-1 infection is relatively low [29].

The childhood vaccination program has had substantial impact on the control of many infectious diseases that were once common in the world. Recently, the HPV vaccination program was introduced into the childhood vaccination progarm as an adolescent-only vaccine being targeted mainly at young girls prior to adolescence and age of onset of sexual activity [30]. The recent HPV immunization routine program is recommended for girls aged 9–14 years in Canada, girls aged 11–12 years in USA, aged 12–13 in UK and girls aged 12 in Italy [30]. Incorporating a vaccine against HSV-2 in this program could be done if it is deemed beneficial.

In this paper, we develop a mathematical model to evaluate the effectiveness of a vaccination program against HSV-2. We focus our study on a vaccination program targeting female school children aged 12–14 years. Our model also includes age structure and gender differences in transmission. We also include varying efficacies of the vaccine against HSV-2, which may not be effective in individuals that are HSV-1 positive. The details of our model and model assumptions are outlined in the next section.

## Methods

**Figure 1:**
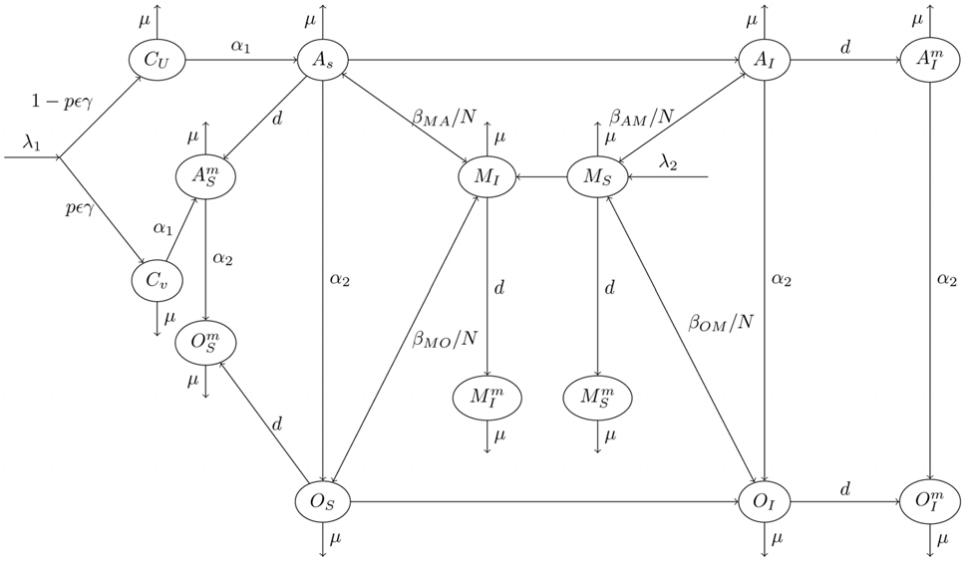
Disease transmission diagram.

The mathematical model, shown in Fig. 1, illustrates how genital herpes can spread in a population. The population is divided into compartments depending on gender (male or female), disease status (infected or uninfected), and susceptibility to the virus (superscript 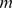 denotes protection). The female population is then further divided according to age (children 13–16 years old (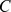), and adults between 16–30 years of age (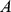) and over 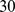 years of age (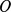)) [22], [23] and vaccination status (vaccinated or unvaccinated). Note that age stratification is only applied to the female population since an adolescent vaccination program for females only, similar to the current HPV vaccination programs adopted in many countries around the world, is being considered.

The mathematical model can be described as follows: female children (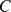) enter the population at rate 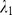. Female children that are HSV-1 seronegative (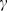), vaccinated (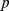) and acquire immunity from the vaccine (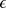) move to the vaccinated/protected class 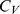 and all others remain in the non-immune class (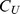). Since several vaccine trails have shown some success in preventing disease, but only in females that are HSV-1 and -2 negative, we incorporate here the HSV-1 seronegative proportion 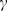 to account for this possible vaccine limitation [27]. It is assumed that vaccination induces lifelong immunity. The non-immune and immune female children (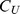 and 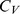 respectively) proceed to the susceptible and protected adult female classes 16–30 years old (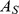 and 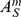 respectively) at rate 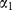 and these individuals mature at rate 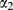 to the older female susceptible and protected classes aged 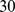 years and older (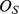 and 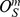 respectively). It is assumed that susceptible females (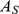 and 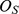) also proceed (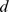) to the protected classes (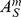 and 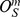) reflecting a change in sexual activity in which they leave the at risk class (i.e. marriage). Susceptible females 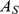 and 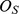 are infected with HSV-2 by infected men (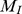) at rates 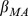 and 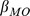 respectively. Infected females 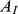 and 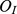 can then, in turn, infected susceptible males (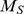) at rates 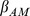 and 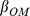 respectively. Infected adult females (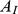) mature at rate 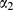 to the older female infected class (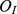). Like the susceptible females (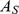 and 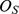), infected females (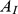 and 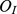), susceptible males (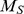) and infected males (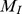) can also change sexual behaviour and move to the 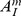, 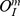, 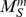 and 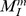 classes respectively. Children and adults die at rate 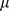 and susceptible males enter the population at rate 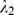. The model equations are written in the Appendix S1.

It is assumed that the incidence rates (the rate of new infections) are proportional to the transmission probability of HSV-2 per sexual partnership and the average number of new sex partners acquired per year, but are inversely proportional to total number of sexually active population [31], [32]. We further assume that female individuals who are not covered by the vaccine program have the same rate of transmission as those who are vaccinated but do not gain immunity against infection. This assumption is based on the following two observations: (1) vaccinated females may have a greater level of sexual activity (higher 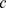) because they feel protected from infection [33]; (2) infectivity during outbreaks may be reduced and outbreaks may occur less often in vaccinated individuals (lower 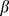) [34].

We model latent and infectious populations together in this framework. The inclusion of latent infections in the infected compartment is reflected by a low transmission rate (see Appendix S1). This allows for a more attractive model for analytical study.

### Parameter Values

Parameter values are listed in Table 1 and are chosen so that they agree with current Canadian and North American statistics, and data reported in the medical literature. The mortality rate is assumed to be approximately 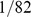 years^−1^, where 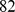 years is the mean life expectancy of Canadians between 2000–2006 [35]. It is assumed that the vaccine will only be effective in 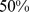 of the female children aged 13 years, which are HSV-1/HSV-2 seronegative (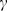) [36]. Vaccine uptake (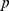) is assumed to lie in the range of 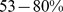 which is similar to the range reported for the adolescent vaccination program against HPV [37]. Immunogenecity (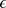) is variable (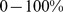).

**Table 1:** List of notations and symbols.

Symbol	Definition	Parameter	Parameter	Reference
		value used	range	
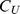	Non-immune females aged 12 years			
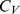	Vaccinated girls with immunity			
	aged 12 years			
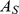	Susceptible females aged 16–30 years			
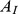	Infected females aged 16–30 years			
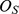	Susceptible females aged over 30 years			
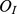	Infected females aged over 30 years			
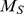	Susceptible males			
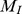	Infected males			
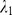	Female population birth rate	27		calculated
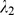	Male population birth rate	30		calculated
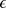	Efficacy of vaccine in girls		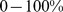	
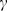	Proportion of females by age 12 that are	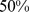	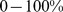	[36]
	HSV-1 and HSV-2 seronegative			
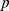	Proportion of females vaccinated	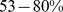	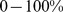	[37]
	with HSV-2 vaccine by age 12			
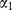	Maturation rate (per year) from children	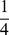	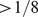	calculated
	to young adults			
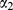	Leaving rate (per year) from young adults	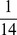		[22], [23]
	to older adults			
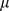	Mortality rate (per year)	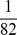		[35]
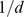	Average length of sexual life span (i.e. time	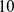	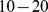	[39]
	span for acquiring new sex partners) (years)			
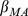	Transmission rate (per year) from an infected male	2.82		calculated
	to a female (16–30 years of age)			[22], [25], [36], [43]
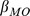	Transmission rate (per year) from an infected male	3		calculated
	to a female (over 30 years of age)			[22], [25], [36], [43]
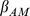	Transmission rate (per year) from an infected female	2.52		calculated
	(16–30 years of age) to a male			[22], [25], [36], [43]
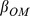	Transmission rate (per year) from an infected female	2.58		calculated
	(over 30 years of age) to a male			[22], [25], [36], [43]

It is assumed that the average age of progression of female children to sexual activity (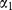) is between 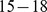 years [38], that young adult females progress to the older adult female class at rate 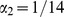 years^−1^ (see [22], [23]), and that adult females and males remain in the ‘effective’ sexually active population for an average of 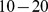 years (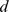, time span for selecting new partners) [39]. This agrees with statistics from the National Survey of Family Growth [40] and the Canadian Community Health survey [41] whereby individuals 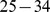 years of age have a higher probability of entering into monogamous relationships, and that the probability of sexual activity out of these monogamous relationships is small.

Similar to [42], we calculate the maturation rate of female children to sexually active young adults:
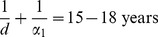
where 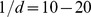 years [39] and thus, 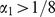 years^−1^.

Females have a greater risk of acquiring genital herpes from male partners than males do from female partners [22], [25]. It is assumed that 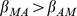 and 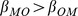, where the 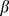
's are related to transmission probabilities per sexual partnership and the average number of sex partners by age. Parameters are chosen so that the prevalence of HSV-2 in men and women is about 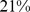, in line with Canadian and North American estimates [22], [36], [43].

## Results

### Control Threshold

The basic reproductive ratio, 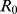, defined as a the number of secondary infections produced by a single infective in a totally susceptible population, is a quantity that determines whether a given disease may spread, or die out in a population. If 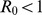 then the disease will die out, however, if 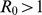 then it will increase in the population. A second quantity called the control reproductive ratio, 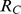, can be used to determine whether a control policy, such as vaccination, will be successful in reducing the number of secondary infections to be less than one. For a review of methods to calculate 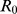, see [44].

Applying the survival function method [44], [45], 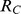 can be rewritten as:
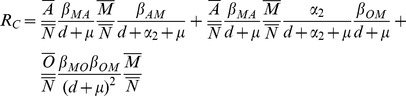
(1)and
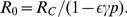
(2)The threshold 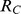 can be explained relative to the underlying biology as follows: when an infective male is introduced into the population, new infected males can be made through three possible pathways:
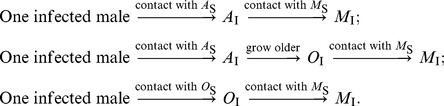
which correspond to the first, second and third terms of Eq. (1) respectively. For instance, using the second path, we see that when one infected male is introduced into the population, the male produces, on average, 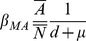 infected young adult females aged 16–30 years during his average sexual lifespan (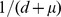). These young adult females then grow into adult females aged 30 years and older over their lifespan 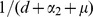 at a rate 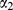 and then each infected older adult female produces, on average, 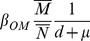 infected males during her sexual lifespan 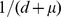. Thus through the second path, the average number of new infected males produced by a single infected male in a population consisting only of susceptible females and males is 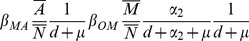. With the parameter values listed in Table 1, the basic reproduction number 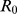 is 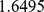, that is, one infected male makes 1.65 new infected males (or 1 infected female makes 1.65 new infected females) when this individual is introduced into a population composed only of susceptible males and females. If we assume the parameters related to a vaccine program are 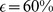, 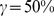, and 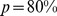, then the reproduction number 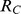 becomes 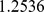.

Note that a reduction in the infectivity (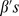) will also decrease 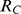. Moreover, in order to eradicate HSV-2 from a population, the vaccination program must be effective in reducing 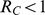 (see Theorem A3 in Appendix S1 for theoretical results). From Eq. (2) we find that this will occur when a critical vaccination threshold (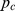) is achieved, where
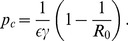
(3)


### Numerical Simulations

**Figure 2:**
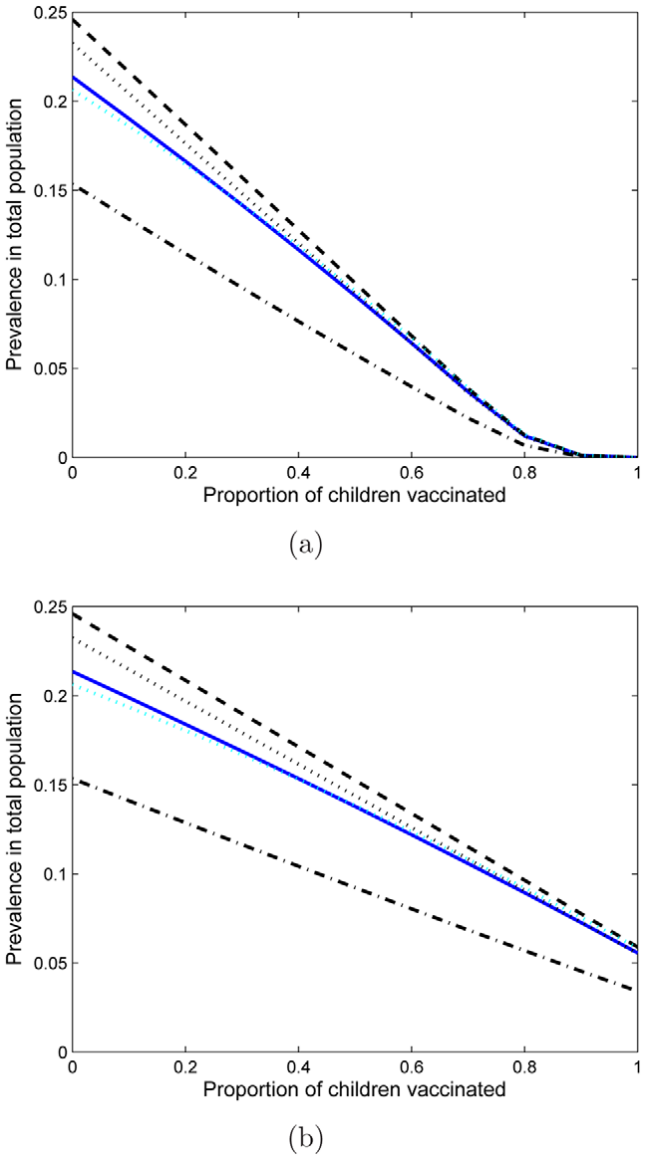
Prevalence of HSV-2 with different levels of vaccination. Prevalence of HSV-2 decreases as vaccine uptake increases in the total population (solid line), women ages 16–30 years (dot-dashed line), women ages over 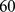 years (dashed line), women ages over 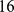 years (black dotted line) and men (gray dotted line). Vaccine efficacy (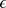) levels are assumed to be 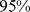 (a) and 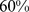 (b) respectively. The proportion of females by age 12 that are HSV-1 and HSV-2 seronegative (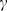) is assumed to be 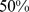.

**Figure 3:**
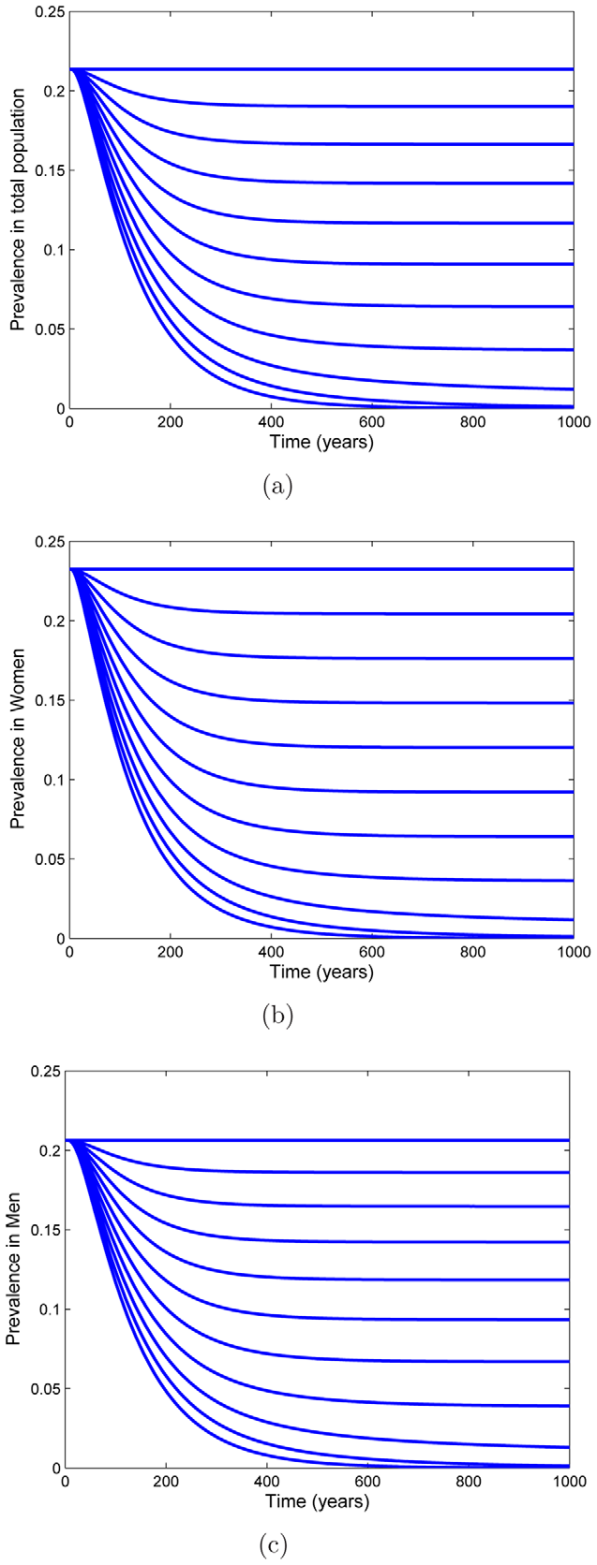
Prevalence of infection over time. Prevalence of infection in the total population (top), women ages over 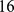 years (bottom left) and men (bottom right) are shown when vaccination is started at year 1 and is given for 999 years. Vaccination levels, from top to bottom, are 0, 10, 20, 30, 40, 50, 60, 70, 80, 90 and 100%. Vaccine efficacy (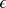) is assumed to be 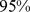 and the proportion of females by age 12 that are HSV-1 and HSV-2 seronegative (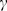) is assumed to be 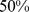.

**Figure 4:**
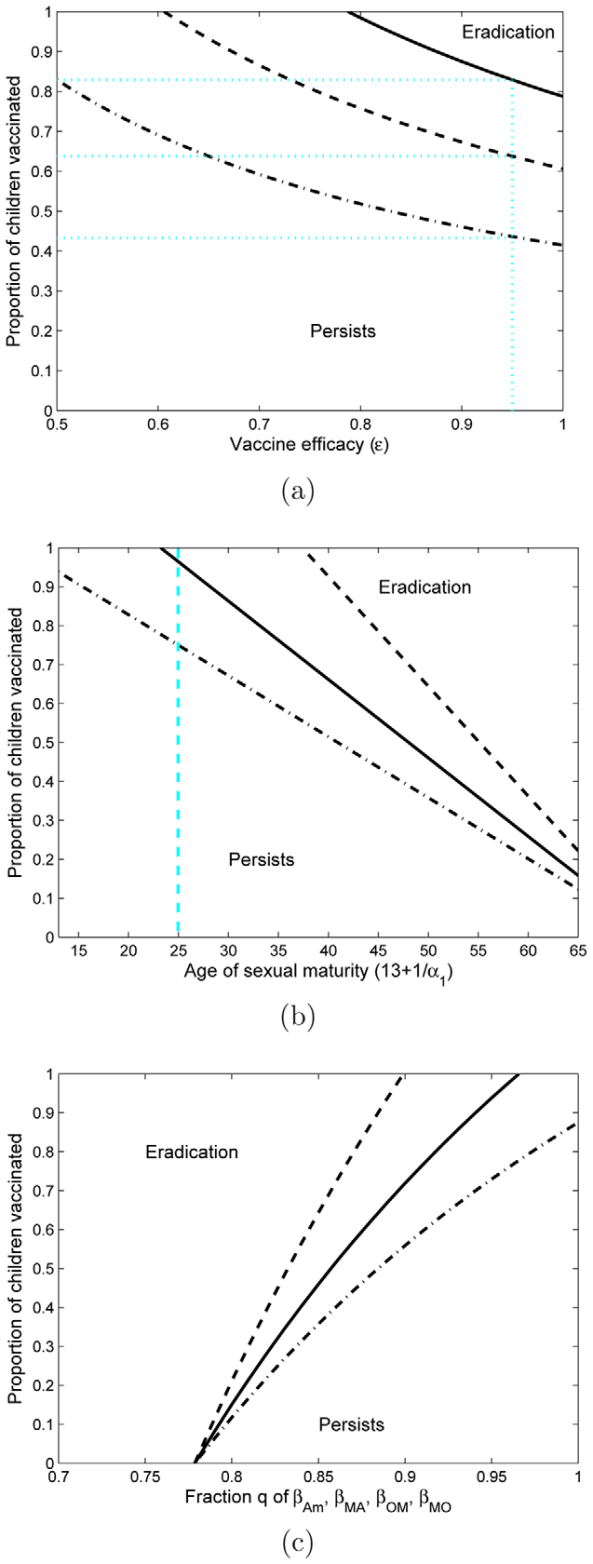
Thresholds of vaccination. Vaccination thresholds for eradication 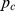 depend on the efficacy of the vaccine (top), age of sexual maturity (bottom left) and rate of transmission (bottom right). (a) Threshold curves depending on vaccine efficacy for three levels of HSV-1 and HSV-2 negative children (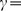
50, 65 and 95%, solid line, dashed line, dot-dashed line respectively) at the time of vaccination. All lines denote threshold corresponding to a vaccine immunogenecity of 95%. (b) Threshold curves depending on age of sexual maturity for three levels of vaccine efficacy (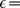
50, 70 and 90%, dashed line, solid line, dot-dashed line respectively) The vertical line at age 25 represents the maximum age at which age of sexual maturity could be achieved to sustain a population. (c) Threshold curves depending on reduced transmission rates (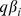, 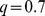 to 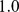) for three levels of vaccine efficacy (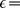
50, 70 and 90%, dashed line, solid line, dot-dashed line respectively).

An adolescent-only vaccination program may be effective in eliminating HSV-2 (Fig. 2). However, regardless of the vaccine efficacy and uptake, the time course of eradication would take many decades (Fig. 3). Also eradication may not result (Fig. 3). For similar vaccination uptake to that of the HPV vaccination program (53–80%), HSV-2 elimination may not be achieved (Fig. 4a, solid line). If vaccine efficacy is 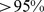, or if the proportion of children that are HSV-1/HSV-2 negative decreases (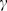), then eradication is more likely, but high vaccination uptake is still required (Fig. 4a). An important, if commonsense, conclusion is that vaccination of some females reduces infection in men, which then reduces infection in women (Fig. 3b,c).

To aid in the reduction of HSV-2 prevalence, education programs about genital herpes may be implemented. These programs will have an important effect if vaccination uptake or if the efficacy of the vaccine is low. A possible effect of these programs is an increase in the average age of progression of children to sexual activity. Fig. 4b demonstrates that as the average age of sexual maturity (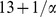) increases, the proportion of females needed to vaccinate to eradicate HSV-2 decreases. However, eradication without vaccination would not be feasible since it is impossible for the age of sexual maturity to be high (Fig. 4b) as populations would not be sustained. Also, even with high levels of efficacy (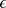) vaccination is unlikely to eradicate HSV-2 since very high levels of vaccine uptake, which are greater than what is seen in HPV, are required.

Another possible effect of education programs is an increase in safe sex practices. This includes the use of condoms, and can include the use of antivirals in those infected with HSV-2. The use of condoms have been routinely recommended for the prevention of transmission of sexually transmitted diseases such as HSV-2 [12], especially in the absence of vaccine. The correct and consistent use of condoms can greatly reduce a person's risk of acquiring HSV-2 [12]. Daily suppressive therapy can also reduce the risk of HSV-2 transmission by up to 50% [46]. Fig. 4c demonstrates that if safe sex practices reduce infectivity (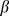
's) to 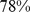 or lower then HSV-2 can be eradicated. However, this result is hampered by the fact that genital herpes can be transmitted even when condoms and/or antivirals are use [46]. Also, this figure assumes that safe sex practices are equally effective in the prevention of infection of females and males. Therefore, the vaccination coverage reported here is simply a lower bound that needs to be achieved in a population where condoms are consistently used.

**Figure 5:**
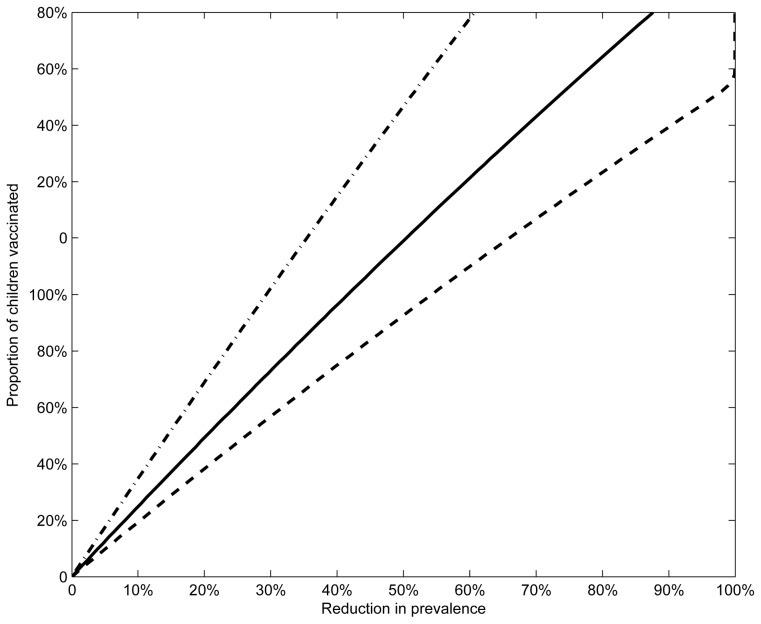
Reduction in prevalence. Vaccination thresholds for reduction in prevalence by 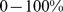 are shown for three different levels of vaccine efficacy (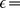 50, 70 and 90%, dashed line, solid line, dot-dashed line respectively).

From the above examples it was shown that eradiction of HSV-2 will be very difficult to obtain. The goal of the vaccination program can then be to decrease disease prevalence in the population. Fig. 5 shows the proportion of children needed to vaccinate so that the prevalence of infection can be reduced by 0–100%, for three different levels of vaccine efficacy. This figure demonstrates that for similar vaccination uptake to that of the HPV vaccination program (53–80%), HSV-2 prevalence can be reduced by 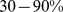, however, this reduction in prevalence can still take many decades to achieve (Fig. 3).

Pregnant females infected with genital HSV-2 (particularly those with a primary infection) can transmit infection to the neonate, which can lead to serious neonatal complications, such as neurologic problems and even death. Thus, it is important to consider the impact of vaccination on the prevention of HSV-2 in the child-bearing age group. Fig. 2 shows the prevalence of infection in women over 16–30 years of age before and after vaccination has been introduced (dot-dashed line). Since the majority of pregnant mothers are from this age group, this demonstrates that the adolescent-only vaccination program results in a decrease in the number of infected pregnant females. This will in turn result in a decrease of maternal-fetal transmission of HSV-2 infection.

**Figure 6:**
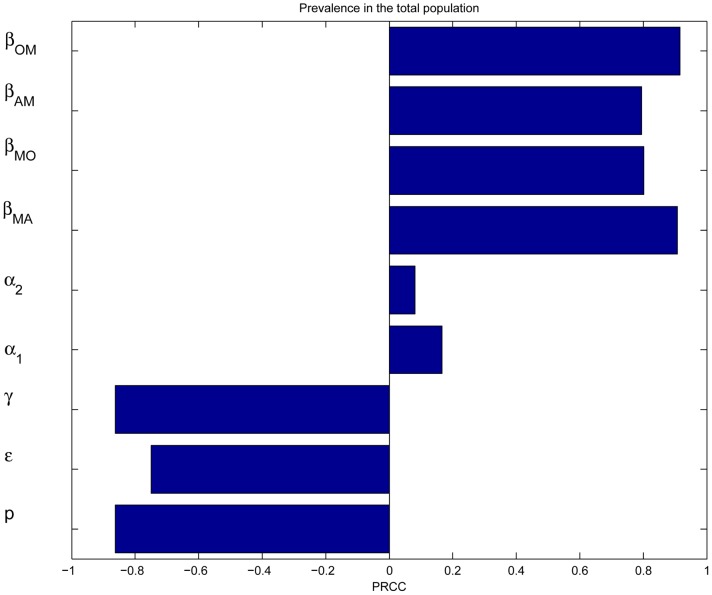
Senstitivity analsysis. The partial rank correlation coefficients are shown using the total population as the index.

Intensive sensitivity analyses can be performed in various ways to determine which parameters play a major role in affecting the predicted results. We perform the sensitivity analysis by using the Latin Hypercube Sampling (LHS) process, which is numerically computed using the method described in [47], [48]. In the LHS method, by using a uniform distribution, values are chosen randomly without replacement from each of the 9 parameters in specified ranges. Following this procedure, for each of these parameters, a partial rank correlation coefficient (PRCC) value is calculated. PRCC values provide a measure of the relationship between model inputs and outputs and are particularly useful for such relationships that are nonlinear but monotonic. PRCC values range between −1 and 1 with the sign determining whether an increase in the parameter value will decrease (−) or increase (+) the specified model output. The PRCC results for disease prevalence in the total popualtion are shown in Fig. 6. This figure indicates that the transmission rates 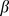 and parameters related to the vaccination programs (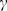, 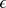, 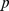) are statistically significant (with 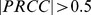). In contrast, the progression rates 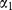 and 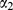 are relatively insignificant. These results highlight the importance of vaccine uptake, vaccine efficacy, HSV-1 and HSV-2 status, and safe sex in the control of genital herpes. The PRCC results for HSV-2 prevalence in men, adult and older women, and the control reproduction number are similar to that shown in Fig. 6.

## Discussion

Genital herpes is one of the most prevalent sexually transmitted diseases in the world. Currently, the development of a vaccine agsint HSV-2, the main cause of genital herpes, is a major focus of study. We have developed a model of HSV-2 transmission that delineates the population by gender and age, two factors that have been shown to be effective predictors of HSV-2 risk, to assess the efficacy of a vaccination program against HSV-2 similar to the vaccination program already implimented against HPV. Using this model we have found that eradication of HSV-2 may be achievable, however, this feasibility of eradication depends on several factors.

Firstly, eradication greatly depends on the rate of vaccine uptake and the efficacy conferred by the vaccine. Vaccine uptake needs to be very high (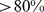) even if the vaccine is very effective in inducing protection against the disease. However, even if vaccine uptake is high, eradication will not be seen for several decades.

The possibility of eradication also depends on education programs that may delay the age of sexual maturity and increase the level of safe sex practices. In these cases, however, vaccination uptake still needs to be very high and may exceed what is actually achievable.

Although eradication will be difficult to achieve, the vaccination program can be effective in reducing disease prevalence to a large extent. Reduction levels of 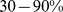 can be achieved, however, these also greatly depend on the vaccine efficacy and the time since the initiation of the vaccination program.

An extension of the current model may be to study the effects of vaccination in age groups less than 14 years of age (and perhaps infants) where HSV-1 prevalence is decreased, and study the effects of a vaccine that is only partially affected by HSV-1 prevalence. Both of these scenarios can be compared to a variation in parameter 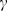. Using the current model we see that the proportion of children needed to vaccinate to eradicate HSV-2 is decreased considerably when 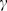 increases.

Another future direction may be to study the effects of booster vaccines in adult females, since vaccine induced immunity to HSV-2 may wane over time. Further delineation of the population including oral infection versus genital infection and sexual preference may also be important when considering the number of booster vaccinations required and the time between them.

A limitation of the current model is we do not model varying rates of virus shedding. HSV-2 infection is actually characterized by rapid episodes of asymptomatic shedding punctuated by increased quantities of virus [2], [49]. Inclusion of varying rates of viral shedding a course for future work.

In this study it is assumed that the HSV-2 vaccine can induce protection in vaccinated individuals, thereby preventing infection. Development of a vaccine against HSV-2 that could provide protective immunity is thought to be unrealistic. The goals in vaccine development against HSV-2 have been to (1) prevent the establishment of latent infection, (2) to reduce the severity of the symptoms, and (3) to reduce the frequency of recurrences [50]. Therefore, our results provide a best case scenario of what could be achieved, and suggest that eradication of HSV-2 would not be possible with vaccines that merely decrease symptoms and the frequency of recurrences, since transmission is still possible in these instances.

## Supporting Information

Appendix S1 **Mathematical analysis of the model.** The mathematical model is formulated as a system of ordinary differential equations. We find the model equilibria, the basic reproductive ratio, and provide some local and global stability analysis of the model. Justification of the SI framework is also provided.
(PDF)
