# Supplementary material for: Epidemiological Impact of a Genital Herpes Type 2 Vaccine for Young Females
Source: PLoS One. 2012 Oct 11;7(10):e46027. doi: 10.1371/journal.pone.0046027 (PMC3469571; doi:10.1371/journal.pone.0046027)
Supplement: Appendix S1 — Mathematical analysis of the model. The mathematical model is formulated as a system of ordinary differential equations. We find the model equilibria, the basic reproductive ratio, and provide some local and global stability analysis of the model. Justification of the SI framework is also provided. (PDF) [file pone.0046027.s001.pdf]

APPENDIX

MATHEMATICAL ANALYSIS OF THE MODEL

A1. MODEL

The full model is listed here. Note that this model includes populations that are not part of the sexually active population at risk of acquiring or transmitting infection i.e. married individuals, vaccinated individuals that are single or married. M_j^m, A_j^m and O_j^m, where $j = S, I$ denote these populations. These populations are needed to correctly calculate the prevalence of infection in the total population.

The mathematical model is as follows:

$$\begin{aligned}
\frac{dC_U}{dt} &= (1-\varepsilon\gamma p)\lambda_1 - (\mu+\alpha_1)C_U \ , \\
\frac{dC_V}{dt} &= \varepsilon\gamma p\lambda_1 - (\mu+\alpha_1)C_V \ , \\
\frac{dA_S}{dt} &= \alpha_1 C_U - \beta_{MA}A_S M_I/N - (d+\alpha_2+\mu)A_S \ , \\
\frac{dA_I}{dt} &= \beta_{MA}A_S M_I/N - (d+\alpha_2+\mu)A_I \ , \\
\frac{dO_S}{dt} &= \alpha_2 A_S - \beta_{MO}O_S M_I/N - (d+\mu)O_S \ , \\
\frac{dO_I}{dt} &= \alpha_2 A_I + \beta_{MO}O_S M_I/N - (d+\mu)O_I \ , \\
\frac{dA_S^m}{dt} &= dA_S + \alpha_1 C_v - (\mu+\alpha_2)A_S^m \ , \\
\frac{dA_I^m}{dt} &= dA_I - (\mu+\alpha_2)A_I^m \ , \\
\frac{dO_S^m}{dt} &= dO_S + \alpha_2 A_S^m - \mu O_S^m, \\
\frac{dO_I^m}{dt} &= dO_I + \alpha_2 A_I^m - \mu O_I^m, \\
\frac{dM_S}{dt} &= \lambda_2 - \beta_{AM}M_S A_I/N - \beta_{OM}M_S O_I/N - (d+\mu)M_S \\
\frac{dM_I}{dt} &= \beta_{AM}M_S A_I/N + \beta_{OM}M_S O_I/N - (d+\mu)M_I \ , \\
\frac{dM_S^m}{dt} &= dM_S - \mu M_S^m \\
\frac{dM_I^m}{dt} &= dM_I - \mu M_I^m.
\end{aligned} \tag{A1}$$

We obtain the equation for the total population size by summing the equations for the population subclasses:

$$N' = (\lambda_1+\lambda_2) - \mu N.$$

This equation has the solution $N(t) = (\lambda_1+\lambda_2)/\mu + (N(0) - (\lambda_1+\lambda_2)/\mu)e^{-bt}$. Thus, the total population size at equilibrium is $\overline{N} = (\lambda_1+\lambda_2)/\mu$ as time evolves. In the following, we will discuss the mathematical analysis of the model (A1).

We can see from $\dfrac{dC_U}{dt} = (1-\varepsilon\gamma p)\lambda_1 - (\mu+\alpha_1)C_U$ that $\lim_{t\to\infty} C_u(t) = (1-\varepsilon\gamma p)\lambda_1/(\mu+\alpha_1)$. Since $\dfrac{d(A_S+A_I)}{dt} = \alpha_1 C_U - (d+\alpha_2+\mu)(A_S+A_I)$, which is asymptotic to $\dfrac{d(A_S+A_I)}{dt} = \alpha_1(1-\varepsilon\gamma p)\lambda_1/(\mu+\alpha_1) - (d+\alpha_2+\mu)(A_S+A_I)$, the asymptotically autonomous system theorem [1] implies $\lim_{t\to\infty}(A_S(t)+A_I(t)) = \alpha_1(1-\varepsilon\gamma p)\lambda_1/((\mu+\alpha_1)(d+\mu+\alpha_2))$. Using a similar approach, we can show the following asymptotic behaviors of solutions:

$$\begin{aligned} & \lim_{t\to\infty}(C_U, C_V, A_S+A_I, O_S+O_I, A_S^m+A_I^m, O_S^m+O_I^m, M_S+M_I, M_S^m+M_I^m, N) \\ = \ & (\overline{C}_U, \overline{C}_V, \overline{A}, \overline{O}, \overline{A^m}, \overline{O^m}, \overline{M}, \overline{M^m}, \overline{N}), \end{aligned} \tag{A2}$$

where $\overline{C}_U = (1-\varepsilon\gamma p)\lambda_1/(\mu+\alpha_1)$, $\overline{C}_V = \varepsilon\gamma p\lambda_1/(\mu+\alpha_1)$, $\overline{A} = \alpha_1\overline{C}_U/(d+\alpha_2+\mu)$, $\overline{O} = \alpha_2\overline{A}/(d+\mu)$, $\overline{A^m} = (d\overline{A}+\alpha_1\overline{C}_V)/(\mu+\alpha_2)$, $\overline{O^m} = (d\overline{O}+\alpha_2\overline{A^m})/\mu$, $\overline{M} = \lambda_2/(d+\mu)$, $\overline{M^m} = d\overline{M}/\mu$, $\overline{N} = (\lambda_1+\lambda_2)/\mu$.

Therefore, we have the following asymptotic system for the infectious classes

$$\begin{aligned} \frac{dA_I}{dt} &= \beta_{MA}(\overline{A}-M_I)M_I/\overline{N} - (d+\alpha_2+\mu)A_I \ , \\ \frac{dO_I}{dt} &= \alpha_2 A_I + \beta_{MO}(\overline{O}-O_I)M_I/\overline{N} - (d+\mu)O_I \ , \\ \frac{dA_I^m}{dt} &= dA_I - (\mu+\alpha_2)A_I^m \ , \\ \frac{dO_I^m}{dt} &= dO_I + \alpha_2 A_I^m - \mu O_I^m, \\ \frac{dM_I}{dt} &= \beta_{AM}(\overline{M}-M_I)A_I/\overline{N} + \beta_{OM}(\overline{M}-M_I)O_I/\overline{N} - (d+\mu)M_I \ , \\ \frac{dM_I^m}{dt} &= dM_I - \mu M_I^m. \end{aligned} \tag{A3}$$

Since the equations for A_I^m, O_I^m and M_I^m can be omitted from the above system, we will first focus on the stability analysis of the following decoupled system

$$\begin{aligned} \frac{dA_I}{dt} &= \beta_{MA}(\overline{A}-M_I)M_I/\overline{N} - (d+\alpha_2+\mu)A_I \ , \\ \frac{dO_I}{dt} &= \alpha_2 A_I + \beta_{MO}(\overline{O}-O_I)M_I/\overline{N} - (d+\mu)O_I \ , \\ \frac{dM_I}{dt} &= \beta_{AM}(\overline{M}-M_I)A_I/\overline{N} + \beta_{OM}(\overline{M}-M_I)O_I/\overline{N} - (d+\mu)M_I. \end{aligned} \tag{A4}$$

Denote $R_C = \beta_{MA}\frac{\overline{A}}{\overline{N}}\frac{1}{d+\mu}\beta_{AM}\frac{\overline{M}}{\overline{N}}\frac{1}{d+\alpha_2+\mu} + \beta_{MA}\frac{\overline{A}}{\overline{N}}\frac{1}{d+\mu}\beta_{OM}\frac{\overline{M}}{\overline{N}}\frac{\alpha_2}{d+\alpha_2+\mu}\frac{1}{d+\mu} + \beta_{MO}\frac{\overline{O}}{\overline{N}}\frac{1}{(d+\mu)^2}\beta_{OM}\frac{\overline{M}}{\overline{N}}$. If we linearize the system (A4) at zero, then the characteristic equation of the linearized system is

$$\lambda^3 + B_1\lambda^2 + B_2\lambda + B_3 = 0,$$

where

$$\begin{aligned} B_1 &= 2(d+\mu) + (d+\alpha_2+\mu), \\ B_2 &= 2(d+\mu)(d+\alpha_2+\mu) + (d+\mu)^2 - \beta_{MO}\frac{\overline{O}}{\overline{N}}\beta_{OM}\frac{\overline{M}}{\overline{N}} - \beta_{MA}\frac{\overline{A}}{\overline{N}}\beta_{AM}\frac{\overline{M}}{\overline{N}}, \\ B_3 &= (d+\mu)^2(d+\alpha_2+\mu)(1-R_C). \end{aligned}$$

By the Routh-Hurwitz condition, all eigenvalues have negative real part if $B_1 > 0$, $B_3 > 0$ and $B_1B_2 - B_3 > 0$. Here, B_1 is always positive, and $B_3 > 0$ if and only if $R_C < 1$. Thus the local stability of the zero equilibrium of (A4) is given by the following lemma.

Lemma A1. *(i) If $R_C < 1$, then the zero equilibrium is locally asymptotically stable.*

(ii) If $R_C > 1$, then the zero equilibrium is unstable.

Since the system (A4) is a cooperative system in the biologically feasible region

$$\Gamma \quad := \quad \left\{(A_I, O_I, M_I) \in \mathbb{R}^3_+ : A_I \leq \overline{A}, O_I \leq \overline{O}, M_I \leq \overline{M}\right\}$$

it then follows from [4, Corollary 3.2] that the subsequent result holds.

Lemma A2. *The following statements are valid:*

(i) If $R_C \leq 1$, the trivial equilibrium $(0,0,0)$ is globally asymptotically stable for system (A4) in Γ.

(ii) If $R_C > 1$, the positive equilibrium (A_I^*, O_I^*, M_I^*) is globally asymptotically stable for system (A4) in $\Gamma \setminus \{(0,0,0)\}$.

According to the Lemma A2, we can see that if $R_C \leq 1$, system (A1) has a unique equilibrium, the disease-free equilibrium P_0. If $R_C > 1$, then system (A1) has a positive endemic equilibrium P^*. Moreover, using the theory of internally chain transitive sets (see, e.g., [1, 3])), as processed in [2], we can further show that R_C plays a key role in determining the global dynamics of the whole system:

Theorem A3.

(i) If $R_C \leq 1$, then the disease-free equilibrium P_0 is globally asymptotically stable in $\mathbb{R}^+_{14}$.

(ii) If $R_C > 1$, then the disease-endemic equilibrium P^* is globally asymptotically stable to all nontrivial solutions.

JUSTIFICATION OF THE USE OF SI FRAMEWORK

In this section, we compare two general models, an SI model (Susceptible-Infected) and a SIL model (Susceptible-Infected-Latent), to justify the use of an SI model framework in describing the transmission of genital herpes. A simple SI model is

$$\begin{aligned} \frac{dS}{dt} &= \lambda - \beta_1 SI/N - \mu S, \\ \frac{dI}{dt} &= \beta_1 SI/N - \mu I, \end{aligned} \tag{A5}$$

where $N = S + I$. An SIL framework to describe the genital herpes transmission is

$$\begin{aligned} \frac{dS}{dt} &= \lambda - \beta_2 SI/N - \mu S, \\ \frac{dI}{dt} &= \beta_2 SI/N - (\mu + \alpha_1)I + \alpha_2 L, \\ \frac{dL}{dt} &= \alpha_1 I - \alpha_2 L, \end{aligned} \tag{A6}$$

where $N = S+I+L$. In this system, α_1 and α_2 are the transfer rates between the I and L classes. The total population sizes for both models is λ/μ. For the SI model (A5) the basic reproduction number is $R_1 = \beta_1/\mu$. For the SIL model (A6) the basic reproduction number is $R_2 = ((\mu + \alpha_2)\beta + \alpha_1\alpha_2)/((\mu + \alpha_1)(\mu + \alpha_2))$. These two models have the same longterm behaviour:

Lemma A4. *(i) If the basic reproduction number is less than one, the disease will die out.*

(ii) If the basic reproduction number is greater than one, the disease will remain persistence and there is a positive endemic equilibrium.

When the reproduction number is greater than one, then the infectious population size of the (A5) is $I_1 = (1 - \frac{\mu}{\beta_1})\frac{\lambda}{\mu}$ while that of the (A6) model is $I_2 = \frac{\beta_2(\alpha_2+\mu) - \mu(\alpha_1+\alpha_2+\mu)}{\beta_2(\alpha_1+\alpha_2+\mu)}\frac{\lambda}{\mu}$. To use model (A5) to approximate (A6), we set $I_1 = I_2$. Then we obtain a relationship between β_1 and β_2:

$$\beta_1 = \frac{\mu\beta_2(\alpha_1 + \alpha_2 + \mu)}{\beta_2\alpha_1 + \mu(\alpha_1 + \alpha_2 + \mu)} < \beta_2.$$

Therefore, we may estimate the transmission rate for the SI model from SIL model, while using the SI scheme to describe the disease transmission.

References

[1] **Hirsch MW**, Smith HL, Zhao XQ. Chain transitivity, attractivity, and strong repellors for semidynamical systems. J Dynam Differential Equations 2001; 17:107-131.

[2] **Lou Y**, Zhao XQ. Modelling malaria control by introduction of larvivorous fish. Bull Math Biol. 2011; 73:2384-2407.

[1] **Thieme HR**, Convergence results and a Poincare-Bendixson trichotomy for asymptotically autonomous differential equations. J. Math. Biol. 1992; 30:755-763.

[3] **Zhao XQ**. Dynamical Systems in Population Biology. Springer-Verlag. New York, 2003.

[4] **Zhao XQ**, Jing Z. Global asymptotic behavior in some cooperative systems of functional-differential equations. Canad Appl Math Quart. 1996; 4:421-444.
